# Variation in regional and landscape effects on occupancy of temperate bats in the southeastern U.S.

**DOI:** 10.1371/journal.pone.0206857

**Published:** 2018-11-08

**Authors:** Benjamin D. Neece, Susan C. Loeb, David S. Jachowski

**Affiliations:** 1 Department of Forestry and Environmental Conservation, Clemson University, Clemson, South Carolina, United States of America; 2 U.S. Forest Service, Southern Research Station, Clemson, South Carolina, United States of America; Clemson University, UNITED STATES

## Abstract

Habitat loss, wind energy development, and the disease white-nose syndrome are major threats contributing to declines in bat populations in North America. In the southeastern US in particular, the recent arrival of white-nose syndrome and changes in landscape composition and configuration have driven shifts in bat species populations and distributions. Effective management strategies which address these large-scale, community-level threats require landscape-scale analyses. Our objective was to model the relationship between ecoregional and landscape factors and occupancy by all bat species in South Carolina, USA, during summer. We conducted acoustic surveys from mid-May through July 2015 and 2016 and evaluated temporally dynamic occupancy models for eight bat species or species groups at the 100 km^2^ level. We found significant effects of landscape factors such as ecoregion and forest edge density for three species, but habitat condition effects were not statistically significant for five other species. Thus, for some species, site-use analyses may be more appropriate than larger scale occupancy analyses. However, our occupancy predictions generally matched statewide historical distributions for all species, suggesting our approach could be useful for monitoring landscape-level trends in bat species. Thus, while our scale of study was likely too coarse for assessing fine-scale habitat associations for all bat species, our findings can improve future monitoring efforts, inform conservation priorities, and guide subsequent landscape-scale studies for bat species and community-level responses to global change.

## Introduction

Bats, a diverse and widespread order of mammals that provide important ecosystem services, have been experiencing significant regional declines due to the introduction and spread of disease, wind energy development, and habitat loss [1–6]. Understanding how these threats drive trends in bat populations at a landscape scale allows the development of effective management strategies [7–9]. In particular, human land use change (e.g., urbanization, loss of forest cover, fragmentation, increase in edge density) may influence the presence of bat species due to the importance of roosting and foraging sites for bat reproduction and survival [10–13]. For instance, the presence and activity of many bat species are positively associated with forest cover [14–16], and the loss and alteration of forest cover influences occupancy rates of different habitats [17,18]. Forest fragmentation can also negatively impact bat abundance, distribution, and use of space [19–23] although in some cases forest fragmentation can have a positive effect of bat activity [24]. Other landscape factors associated with urbanization and development may also influence habitat use by bats. Roads can serve as either conduits or barriers to movement, depending on the type of road and the surrounding habitat [25–31]. For example, rural unpaved roads surrounded by forests in Indiana are selected over paved roads surrounded by development and open areas [32]. Roads also act as barriers to movements as well as sources of mortality [33]. Other factors associated with urbanization and loss of forest habitat such as poor water quality and artificial lights may also affect bat habitat use and activity [34–36]. Additionally, climate change is expected to cause widespread changes in land cover and habitat for bats [2].

Shrinking habitat for terrestrial vertebrates has been predicted for the southeastern United States, especially in scenarios with expansion of urban and agricultural areas [37]. For instance, the percentage of developed land in the contiguous United States is predicted to double from 1997 to 2025, with the greatest increase in southern regions [27], leading to significant reductions in wildlife habitat and increased habitat fragmentation. Based on historical land use change, Wear et al. [38] predict urban land coverage to at least double in the southern United States from 1997 to 2060, and predict a 7–13% decline in forest coverage across the south, with the Piedmont region losing the greatest percentage of forest (up to 21%). Loss of forest cover and increasing urbanization are likely to greatly impact the abundance and distribution of bats across the southeast but few data are available to predict how various bat species will respond.

Effective conservation strategies that address various threats to bat populations in the southeastern United States require landscape scale monitoring and analyses [2,39]. Thus, our objective was to conduct an assessment of habitat factors influencing bat species habitat use at the landscape scale, which may provide valuable insight into how predicted land use and land cover change could affect bats. We used temporally dynamic (i.e., multi-season) analyses to estimate effects of habitat metrics on species occupancy rates and calculate annual colonization and extinction rates to account for year-to-year variation for a suite of bat species encountered across South Carolina, USA. We also generated species distribution maps based on our model predictions, which may be used in the future to reveal changes in species distributions in response to changing habitat [9]. Our species-specific findings can also be used to inform landscape management decisions that may affect bat populations, guide subsequent, finer-scale investigations in species-specific patterns of habitat use, and serve as a baseline for future comparative studies examining changes in bat habitat use and species distributions over time.

## Methods

### Ethics statement

This study did not entail capture of vertebrates and relied on non-invasive acoustic sampling that did not alter the behavior of the animal. Thus, it was not subject to IACUC review. Approval to survey on public lands was granted by the Savannah Parks and Recreation Department; Colleton County Planning and Development Department; Charleston County Park and Recreation Commission; Town of Hilton Head Island; the Edisto Island Open Land Trust; Cape Romain National Wildlife Refuge; South Carolina Forestry Commission; South Carolina Department of Natural Resources; U.S. Army Corps of Engineers; Naturaland Trust; Crosswell Elementary School; South Carolina Department of Parks, Recreation, and Tourism; the U.S. Forest Service; and Greenville Water. Permission to survey on private lands was granted by all landowners.

### Study area

We conducted our study throughout South Carolina and within 10 km of the state border in Georgia and North Carolina. Five of the physiographic regions in the southeastern United States occur in a gradient from northwest to southeast in South Carolina: Blue Ridge, Piedmont, Southeastern Plains, Middle Atlantic Coastal Plain, and Southern Coastal Plain [40]. Land use throughout South Carolina includes developed urban areas, silviculture, agriculture, livestock, and undeveloped land [41], but the dominant land cover varies among regions. Forest was the dominant land cover in the Blue Ridge, forest and hay or pasture were the dominant land cover types in the Piedmont, woody wetlands, forest, shrublands, and cultivated crops were the dominant land cover types in the Southeastern Plains and Middle Atlantic Coastal Plain, and herbaceous wetlands, woody wetlands, forest, and open water were the dominant land cover types in the Southern Coastal Plain [41]. Topographic relief and elevation in South Carolina are greatest in the Blue Ridge, with peaks up to 1085 m, and sharply decrease in the central regions, finally becoming low-elevation plains and wetlands near the Atlantic coast.

Fourteen temperate insectivorous bat species are known to occur within South Carolina. During summer, big brown bat (*Eptesicus fuscus*; EPFU), eastern red bat (*Lasiurus borealis*; LABO), Seminole bat (*L*. *seminolus*; LASE), evening bat (*Nycticeius humeralis*; NYHU), tri-colored bat (*Perimyotis subflavus*; PESU), and Mexican free-tailed bat (*Tadarida brasiliensis*; TABR) occur throughout South Carolina. Rafinesque’s big-eared bat (*Corynorhinus rafinesquii*; CORA), northern yellow bat (*Dasypterus intermedius*; DAIN), hoary bat (*L*. *cinereus*; LACI), silver-haired bat (*Lasionycteris noctivagans*; LANO), southeastern myotis (*Myotis austroriparius*; MYAU), eastern small-footed bat (*M*. *leibii*; MYLE), little brown bat (*M*. *lucifugus*; MYLU), and northern long-eared bat (*M*. *septentrionalis*; MYSE) have more limited summer distributions within the state [42,43].

### Sampling design

**Fig 1:**
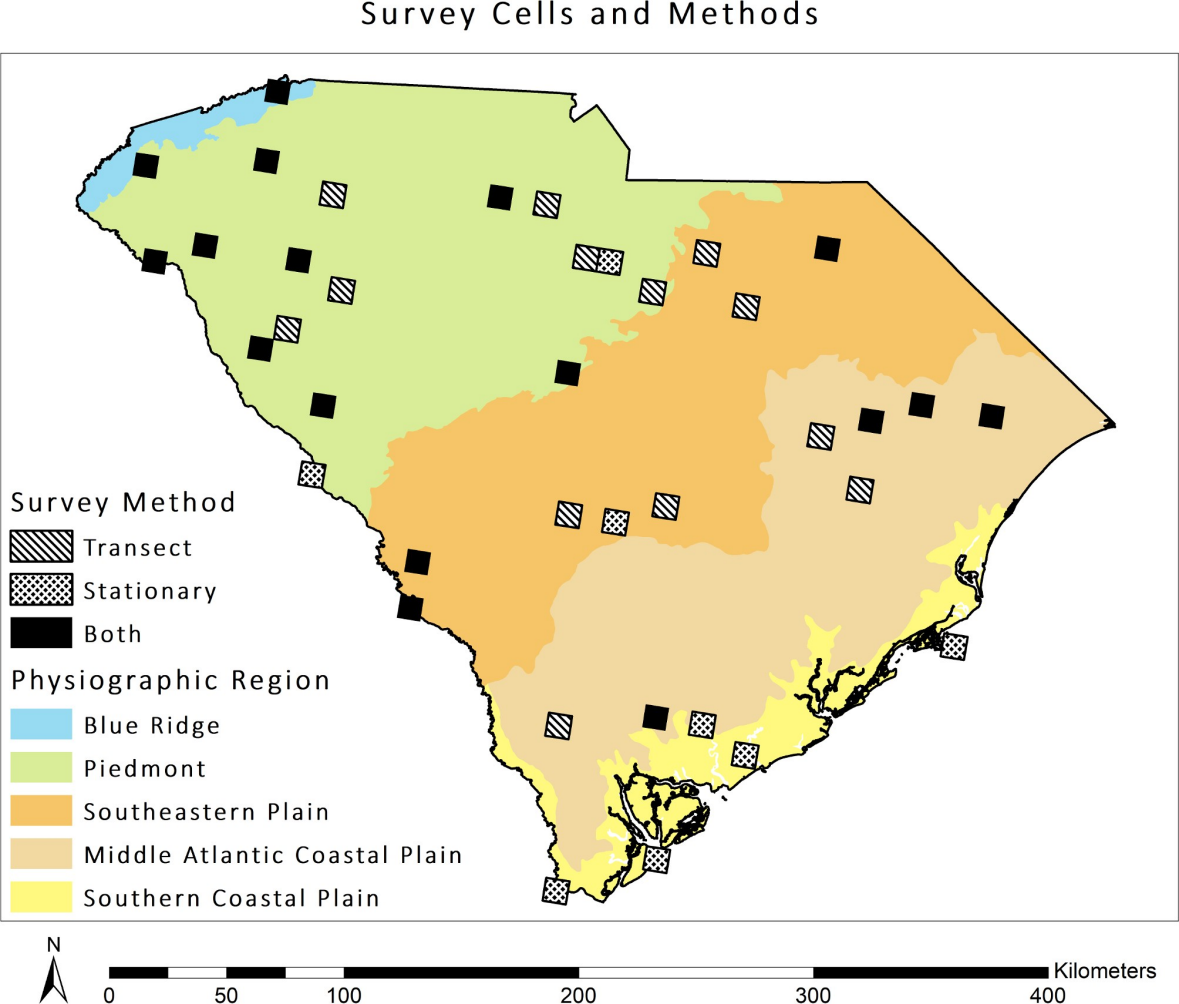
Distribution of NABat priority cells surveyed across South Carolina using mobile transects only, stationary point surveys only, or both survey methods, May-July 2015 and 2016. Physiographic regions of South Carolina are also displayed.

We used the North American Bat Monitoring Program (NABat) framework to acoustically survey bat species across South Carolina. The sampling frame for NABat consists of a continuous grid of 10 x 10 km cells across North America [8]. We identified priority survey cells within South Carolina based on the NABat master sample, which uses the generalized random tessellation stratified algorithm to assign priority numbers to cells to maintain a spatially balanced and randomly distributed sample (Fig 1 and S1 Table). We conducted stationary point surveys for four consecutive nights and mobile transect surveys on two of the four nights from mid-May through mid-July 2015 and 2016. In 2015, we surveyed 35 cells: 15 with mobile transects only, six with stationary point surveys only, and 14 with both survey methods. In 2016, we surveyed the 35 cells from 2015 and three additional cells (one with mobile transects only, and two with stationary points only) for a total of 38 cells surveyed: 13 with mobile transects only, eight with stationary point surveys only, and 17 with both survey methods. We surveyed three cells with stationary point and mobile transect surveys in 2016 which were surveyed with mobile transects only in 2015 (Fig 1). Each stationary point survey began 30 minutes prior to sunset and ended 30 minutes after sunrise, while each mobile transect survey began 45 minutes after sunset and was driven at 32 kph, with duration dependent on the length of the transect (25–48 km). During each year, we surveyed the same stationary point locations and mobile transect routes within each cell where possible.

We used Anabat SD2 bat detectors with directional, stainless steel microphones (Titley Scientific, Columbia, MO, USA) and 2.5 m microphone cables for both survey methods. For stationary point surveys we mounted the microphone inside a water resistant PVC housing and attached it to the top of a 1.8 m high tripod. For mobile transect surveys we placed the microphone at the center of a vehicle’s roof with no waterproof housing.

### Data processing

To determine which species we detected during surveys, we first removed call files containing no bat calls or calls with fewer than three search-phase pulses using a custom noise filter in AnalookW version 4.2.7 (Titley Scientific, Brendale, Australia) and through manual review of each file. We classified the remaining call files collected during 2015 to species using EchoClass version 3.1 and Kaleidoscope Pro version 3.1.5 (Wildlife Acoustics, Inc., Maynard, Massachusetts, USA), and manually vetted all classifications based on reference calls of each species. For the sake of consistency, all calls were vetted by the senior author (BDN) with the concurrence of the second author (SCL). We observed low classification agreement between automated classifiers, so we manually classified all high-quality search phase calls from 2016. Our reference calls were recorded from captured bats which were identified and light-tagged [44]. We aggregated EPFU and LANO calls as EPFULANO, and LABO and LASE calls as LABOLASE because these species have very similar echolocation call structures. We also grouped calls of MYLE, MYLU, and MYSE as MYLELUSE for analyses because we detected them in very few cells, their echolocation calls can be difficult to distinguish from each other, and their foraging habitat preferences are very similar [11,16]. We included *Myotis* calls that could not be identified to species from the Blue Ridge and Piedmont regions in the MYLELUSE group because only these three *Myotis* species occur in these regions [43]. However, we did not include unknown *Myotis* calls if they occurred in the Coastal Plain due to the possible confusion with *M*. *austroriparius*.

### Data analysis

We hypothesized that probability of occupancy would vary by ecoregion for species with limited ranges within our study area, but that it would not vary by ecoregion for species with statewide ranges (Table 1) [43]. Thus, we included a categorical covariate (Region) based on the primary U.S. Level III Ecoregion [40] within each cell.

**Table 1:** Predicted and observed effects of environmental variables on the probability of occupancy for each species surveyed in South Carolina, May-July 2015 and 2016.

Species	Region	Ag	Dev	Forest	F.Wet	Contagion	F.ED	F.Wet.ED	Stream	Pri	Sec	Qua
**DAIN**	**Y | Y**	- | 0	- | 0	NA	+ | 0	0 | 0	NA	+ | 0	+ | 0	- | 0	- | 0	0 | 0
**EPFULANO**	0 | 0	0 | 0	0 | 0	0 | 0	NA	0 | 0	+ | 0	NA	0 | 0	- | 0	0 | 0	+ | 0
**LACI**	Y | 0	0 | 0	- | 0	+ | 0	NA	0 | 0	**0 | -**	NA	+ | +	- | 0	- | 0	0 | 0
**MYAU**	Y | 0	- | 0	- | 0	NA	+ | 0	+ | 0	NA	+ | 0	+ | 0	- | 0	- | 0	0 | 0
**MYLELUSE**	**Y | Y**	- | 0	- | 0	+ | 0	NA	+ | 0	- | 0	NA	+ | 0	- | 0	- | 0	0 | 0
**NYHU**	0 | 0	+ | 0	0 | 0	+ | 0	NA	0 | 0	+ | -	NA	0 | +	- | 0	0 | 0	+ | 0
**PESU**	0 | 0	- | -	- | +	+ | +	NA	0 | 0	+ | 0	NA	+ | 0	- | 0	- | 0	+ | +
**TABR**	0 | 0	+ | 0	+ | 0	- | 0	NA	0 | 0	0 | 0	NA	0 | 0	- | +	0 | +	0 | +

Predicted effects are left of “|” and observed effects are right of “|”. Significant effects on probability of occupancy are indicated by “Y” as an effect of a categorical covariate or “0” as no effect, and “+” as a positive effect, “0” as no effect, or “-” as a negative effect for continuous variables. “NA” indicates we did not test an effect for a species, based on habitat preferences. Effects that were statistically significant are highlighted with gray background. *Dasypterus intermedius* = DAIN; *Eptesicus fuscus* and *Lasionycteris noctivagans* = EPFULANO; *Lasiurus cinereus* = LACI; *Myotis austroriparius* = MYAU; *M*. *leibii*, *M*. *lucifugus*, and *M*. *septentrionalis* = MYLELUSE; *Nycticeius humeralis* = NYHU; *Perimyotis subflavus* = PESU; and *Tadarida brasiliensis* = TABR. LABOSE were not included in the table because they occurred in every cell and we were unable to model occupancy. Region = physiographic region, Ag = percent agriculture (pasture/hay and cultivated crops), Dev = percent of all classes of development, Forest = percent upland forest, F.Wet = percent bottomland forest, Contagion = a measure of dispersion, F.ED = forest edge density, F.Wet.ED = forested wetland edge densty, Stream = total stream length, Pri = length of primary roads, Sec = length of secondary roads, and Qua = length of quaternary roads.

We hypothesized that the effects of land cover types and forest fragmentation on probability of occupancy varied among species, based on species summer roosting and foraging site preferences [11,16,26,45,46] (Table 1). We calculated percent land coverage within each cell from the National Land Cover Database (NLCD 2011) [47] and aggregated “Pasture/Hay” and “Cultivated Crops” as Ag, all classes of development as Dev, upland forest types as Forest, and “Woody Wetlands” for bottomland forest (F.Wet). We used our reclassified NLCD 2011 data as input in Fragstats version 4.2.1 [48] and calculated Contagion (a landscape measure which increases as land cover type interspersion decreases and dispersion increases) and measures of forest and forested wetland edge density (F.ED and F.Wet.ED) within each cell.

We hypothesized that stream length would have a positive effect on occupancy of many bat species because they prefer to forage near streams and riparian areas [16,34,35,49]. Additionally, streams can act as forest edges and may be important sources of water, so we also hypothesized positive effects of increasing stream length on probability of occupancy for species that commonly forage along edges, but may not be explicitly associated with riparian areas (Table 1). We calculated total stream length within each cell using ‘NHDFlowline’ data from the National Hydrology Dataset (NHD) [50].

Due to effects of roads on species presence [25,26,28–31,51], and because different road classes are often associated with different landscapes [52], we hypothesized effects of roads on bat species occupancy would vary based on road type (Table 1). We used National Transport Dataset (NTD) RoadSegment data [53] and U.S. Forest Service Roads [54], and classified roads into four categories, primarily based on Master Address File/Topologically Integrated Geographic Encoding and Referencing Feature Class Code Definitions (https://www.census.gov/geo/reference/mtfcc.html): primary roads included divided highways with access ramps (Pri), secondary roads included highways with intersections (Sec), tertiary roads included single lane rural and city roads (Ter), and quaternary roads included forest access roads (Qua). We then calculated the length of all four road classes within each cell.

Annual turnover rates (i.e., colonization and extinction) at the landscape scale could indicate changes in species range due to land cover changes or regional threats to bat populations. Given that we only monitored for two years, we predicted that it would be unlikely for land cover change to influence turnover rates. However, regionally, the arrival of white-nose syndrome (WNS) is ongoing in the southeastern US. Therefore, we predicted higher turnover rates for MYELUSE and PESU, because they are affected by WNS in the northwestern part of our study area [55]. In addition, the act of migration could influence turnover rates, and we accordingly predicted high turnover rates for LACI because they exhibit migratory behavior [42].

Due to limited access for stationary point surveys and constraints in establishing mobile transects [56], some stationary point locations were near cell edges and short segments of some mobile transects were up to 2.2 km outside cells. Additionally, we may have detected bats within cells that entered from areas outside cells. Thus, similar to other studies [11,17,18,30,35], we buffered cell boundaries by 2.2 km before we measured covariates to include relevant landscape effects.

We used a multi-season Bayesian occupancy modeling approach to evaluate the influence of hypothesized environmental factors on the probability of occupancy for each species and to calculate turnover rates. At the sample unit-level, this approach models the probability of occupancy within each sample unit during each sampling period (in our case, each year) as a temporally autoregressive function of intercept and covariate effects with parameterization for colonization and extinction. At the survey level, this approach models the probability of detection, which is dependent on the presence of the species, as a function of intercept and covariate effects [9]. We treated cells as our sample unit, considered each night at each point or transect as a separate survey occasion, and created presence/non-detection tables for each species on each survey occasion. We never detected CORA and we detected LABOLASE in every cell each year; therefore, we were unable to model occupancy for these species. Because we surveyed each cell within one week each year, we treated populations as closed within years, and open between years and calculated turnover rates for each species between the two years as the probability that an unoccupied cell became occupied (i.e., colonization), and an occupied cell became unoccupied (i.e., extinction). We used non-informative priors, treated all terms as fixed effects, and used JAGS version 4.1.0 (http://mcmc-jags.sourceforge.net/) through package ‘rjags’ [57] in program R version 3.3.3 (https://www.r-project.org/) to fit models. We ran three independent chains of 25,000 iterations, discarded an initial 5,000 iterations as burn-in, and retained every fourth iteration for a total of 18,750 iterations per model. We assumed model convergence when the potential scale reduction factor (i.e., the Brooks-Gelman-Rubin diagnostic; R-hat) of each parameter was < 1.1. We ranked models using the Widely Applicable Information Criterion (WAIC), which we calculated for each model using the package ‘loo’ version 1.1.0 [58], and considered models closely competing when they were within 2.0 WAIC from the top-ranked model [59]. We calculated ΔWAIC from the top-ranked model, then calculated each model’s relative likelihood, and finally calculated model weights to evaluate relative support of the models. We considered a covariate effect significant if the estimated 95% credible intervals did not include zero.

To account for imperfect detection, we modeled detection probabilities for each species independently and modeled occupancy as a function of intercept only. We used nine survey variables (Survey Type, Survey Duration, Vegetation Clutter [no, medium, or high amounts of vegetation within 5 m of the detector], Survey Date, Survey Issue [detector malfunction or incomplete mobile survey due to weather], Temperature, Relative Humidity, Wind Speed, and Rain) to test hypothesized effects of single-term models and additive effects models [56]. We included the covariates from the top-ranked detection model for each species in the occupancy modeling process (S2 Table).

Based on *a priori* hypotheses, we used single-term models and models with additive effects of some covariates to model occupancy by each species (Table 2). We also tested null and global models. Prior to model fitting, we standardized all continuous covariates to have a mean of 0 and standard deviation of 1. We used Pearson’s correlation to test for correlations among covariates and considered those with a Pearson’s |r| > 0.7 as correlated and did not include them in the same model. Pri, Sec, and Ter road classes were correlated with Dev, Sec was correlated with Ter, and F.Wet was correlated with F.Wet.ED (S3 Table). Therefore, Ter was not included in any models. Since DAIN and MYAU are associated with forested wetlands [16,60], we substituted Forest and F.ED with F.Wet and F.Wet.ED, respectively, and omitted F.Wet.ED from the global model for these species because it was significantly correlated with F.Wet.

**Table 2:** Reasoning for 11 *a priori* occupancy models that we tested for each bat species in South Carolina, USA, May-July 2015 and 2016.

Model	Reasoning
*Region*	May be significant for species with limited distributions
*Ag + Dev + Forest/F*.*Wet*	Land cover measures may be good predictors of habitat quality
*Region + Ag + Dev + Forest/F*.*Wet*	Land cover composition can vary within regions
*Contagion*	Some species require continuous tracts of preferred habitat
*F*.*ED/F*.*Wet*.*ED*	Many species forage along edges
*Forest/F*.*Wet + Contagion*	Some species are associated with contiguous tracts of forest cover
*Stream*	Streams often occur at habitat edges, and they may be important sources of drinking water and foraging areas
*Stream + F*.*ED/F*.*Wet*.*ED*	Streams along forest edges may be more important than those in forest interiors or urban and agricultural areas
*Stream + Ag + Dev + Forest/F*.*Wet*	May describe important foraging and roosting habitat
*Pri + Sec + Qua*	Roads may act as edges for foraging and commuting
*Ag + Dev + Forest/F*.*Wet + Qua*	May predict habitat quality and areas for foraging and commuting

We also tested null and global models.

We used two approaches to evaluate model performance. First, we performed k-fold cross-validation to calculate area under the receiver-operating curve (AUC) for the top detection model, and each model with WAIC ≤ the null occupancy model. AUC values range from 0 to 1, where 0.5 indicates no predictive power and 1.0 indicates perfect predictive performance [61]. We randomly partitioned the data five times, with 66% as training datasets and the remainder of the data as testing datasets. We reviewed partitions to be sure each training dataset included at least one cell from each of the five ecoregions, and used the same partitions to evaluate models for each species. We used the package ‘ROCR’ version 1.0.7 [62] to calculate AUC for each model. We also evaluated model performance by comparing known species distributions, based on Menzel et al. [43], to predicted species distributions. To generate predicted range maps, we measured environmental covariates in all 893 10 x 10 km NABat cells (each buffered by 2.2 km) throughout South Carolina and calculated estimated occupancy rates for each cell based on the top-ranked occupancy model for each species. We then compared these predicted occupancy maps to historical range maps for each species based on Menzel et al. [43].

## Results

We recorded 61,397 call files in 2015, 21,972 of which passed our noise filter, and 65,727 call files in 2016, 42,960 of which passed our noise filter. We classified 15,292 and 27,380 call files to species in 2015 and 2016, respectively.

Six of the eight species or species groups had occupancy models that ranked higher than the null model. The null model ranked highest for EPFULANO and MYAU, NYHU had two top-ranked models, and DAIN, LACI, MYLELUSE, and NYHU had at least three models that closely competed with the top-ranked model (i.e., within 2.0 WAIC; Table 3). Ecological models performed better than null models for four of the species, but the predictive performance of the top-ranked models varied, from 0.46 for LACI to 0.76 for NYHU, and was low for many of the models (Table 3). Additionally, DAIN, LACI, MYLELUSE, NYHU, and TABR had top-ranked models with equivalent or lesser predictive performance than competing models (Table 3). All covariates in all occupancy models reached convergence, except the Piedmont region in two models for EPFULANO.

**Table 3:** Occupancy probability model results for each species of bat surveyed using acoustic detectors across South Carolina, May-July 2015 and 2016.

Species	Occupancy Model	WAIC	ΔWAIC	Weight	AUC
**DAIN**	*Region*	122.9	0	0.15	0.65
	*Stream + Ag + Dev + F*.*Wet*	123.3	0.4	0.12	0.58
	*Stream*	123.4	0.5	0.12	0.66
	*Pri + Sec + Qua*	123.6	0.7	0.10	0.52
	*Region + Ag + Dev + F*.*Wet + Contagion*				
	*+ Stream + Qua*	124.1	1.2	0.08	0.65
	*Ag + Dev + F*.*Wet + Qua*	124.1	1.2	0.08	0.52
	*Ag + Dev + F*.*Wet*	124.6	1.7	0.06	0.51
	.	124.6	1.7	0.06	0.53
**EPFULANO**	.	440.0	0	0.12	0.48
**LACI**	*Stream + F*.*ED*	170.8	0	0.39	0.46
	*F*.*ED*	171.8	1	0.24	0.53
	.	172.1	1.3	0.21	0.38
**MYAU**	.	214.7	0	0.24	0.62
**MYLELUSE**	*Region*	81.7	0	0.21	0.62
	*F*.*ED*	82.7	1	0.13	0.35
	.	83.2	1.5	0.10	0.67
**NYHU**	*F*.*ED*	457.4	0	0.12	0.76
	*Stream + F*.*ED*	457.4	0	0.12	0.76
	*Pri + Sec + Qua*	457.7	0.3	0.10	0.68
	.	457.8	0.4	0.10	0.34
**PESU**	*Ag + Dev + Forest + Qua*	467.8	0	0.28	0.71
	*Ag + Dev + Forest*	468.9	1.1	0.16	0.53
	*Stream + Ag + Dev + Forest*	469.1	1.3	0.15	0.52
	*Region + Ag + Dev + Forest*	469.6	1.8	0.11	0.53
	*Region + Ag + Dev + Forest + Contagion*				
	*+ F*.*ED + Stream + Qua*	469.9	2.1	0.10	0.52
	*Stream + F*.*ED*	471.6	3.8	0.04	0.35
	*Forest + Contagion*	472.2	4.4	0.03	0.52
	*F*.*ED*	472.2	4.4	0.03	0.41
	*Pri + Sec + Qua*	472.3	4.5	0.03	0.49
	.	472.4	4.6	0.03	0.48
**TABR**	*Pri + Sec + Qua*	390.9	0	0.29	0.60
	.	392.4	1.5	0.14	0.60

Models are ordered from highest to lowest performance based on WAIC, and only those which performed better than the null model are shown. The null model (i.e., intercept only) is indicated by “.”, and “+” indicates additive effects. Model weights based on WAIC scores, and predictive performance based on area under the receiver operator curve (AUC) are shown. Refer to Table 1 for species code definitions, S4 Table for covariate beta estimates, and S2 Table for detection covariates used in each model.

**Fig 2:**
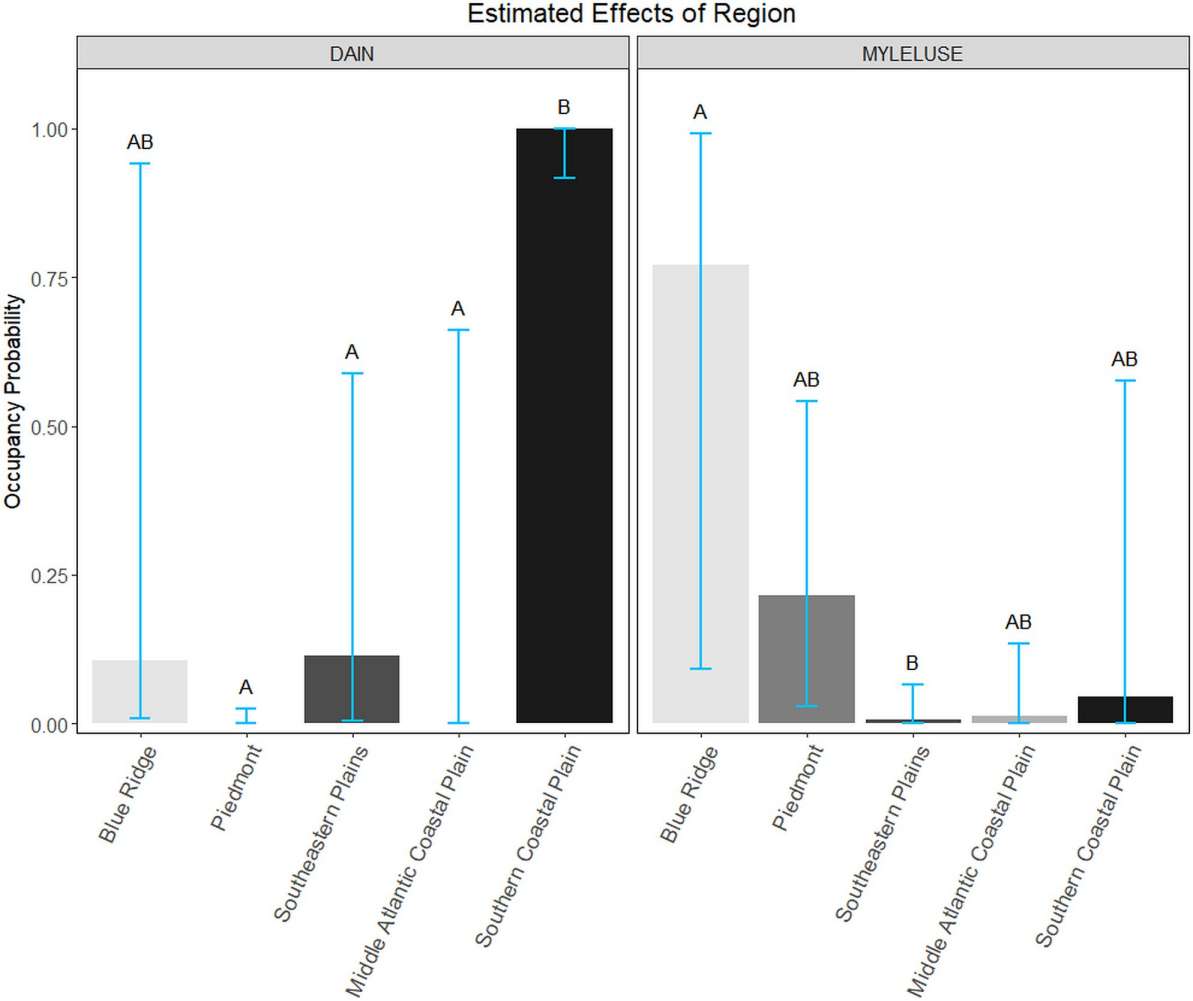
Mean estimated probability of occupancy of northern yellow bats (DAIN) and small-footed bats, little brown bats, and northern long-eared bats combined (MYLELUSE) within each ecoregion of South Carolina, May-July 2015–2016. Blue bars indicate 95% credible intervals. Within species, regions which share a letter above their intervals are not significantly different from one another.

We observed support for the Region model for DAIN and MYLELUSE (Table 3). Mean estimated probability of occupancy for DAIN was significantly higher in the Southern Coastal Plain than all other regions except the Blue Ridge (Fig 2). The mean probability of occupancy for MYLELUSE was highest in the Blue Ridge region, but it only significantly differed from the estimate for the Southeastern Plains (Fig 2).

**Fig 3:**
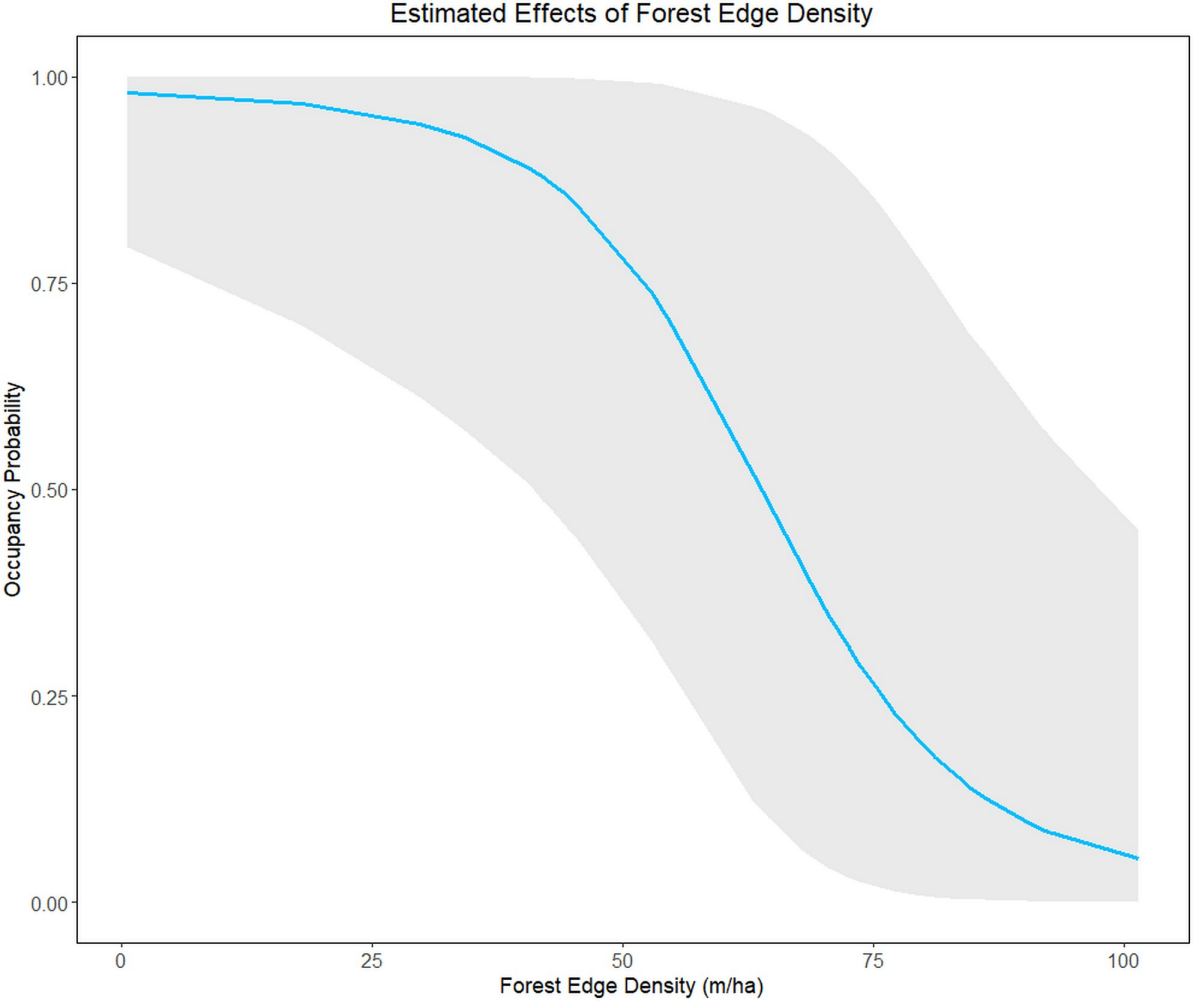
Estimated effect of forest edge density on hoary bat (*Lasiurus cinereus*) probability of occupancy across South Carolina, May-July 2015 and 2016. Probability of occupancy is based on the top ranked model for hoary bats. Gray shading indicates the 95% credible interval.

The Stream + F.ED model was the top-ranked model for LACI and NYHU (Table 3). Forest edge density significantly affected the probability of occupancy for LACI, but did not significantly affect the probability of occupancy for NYHU (Tables 1 and S3). From the lowest (0.50 m/ha) to the highest (101.43 m/ha) forest edge density, LACI probability of occupancy decreased from 98% to 5%, with a steep negative slope beginning at 50 m/ha (Fig 3). Stream length did not significantly affect the probability of occupancy for either species (Tables 1 and S4). In addition to the Stream + F.ED model, we also found equivalent support for the single-term F.ED model for NYHU (Table 3), where the effect of forest edge density was negative but not statistically significant (Tables 1 and S4).

The top ranked model for PESU was Ag + Dev + Forest + Qua (Table 3). Probability of occupancy for PESU was negatively associated with increasing agricultural cover and positively associated with increasing developed land, forest cover, and quaternary road length (Table 1). However, none of these effects were statistically significant (Tables 1 and S4).

We found support for the Pri + Sec + Qua model for TABR (Table 3). Occupancy probability was positively associated with increasing lengths of all road classes (Table 1). Increasing lengths of secondary and quaternary road classes had stronger positive effects on occupancy than the primary road class. However, none of these effects were significant (Tables 1 and S4).

**Fig 4:**
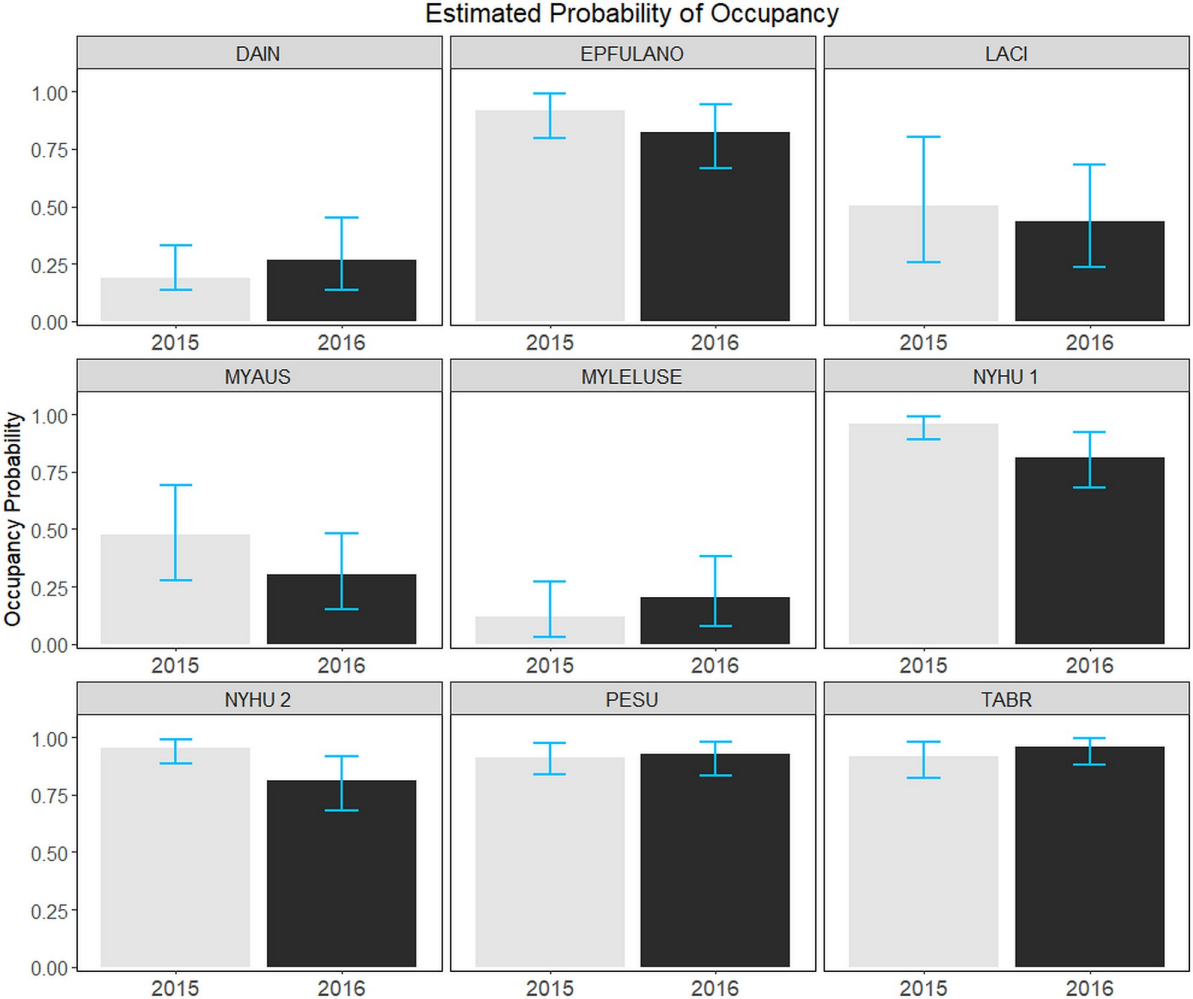
Estimated mean probabilities of occupancy of each species across South Carolina, May-July 2015 and 2016. Estimates are based on the top ranked occupancy model for each species. NYHU had two top-ranked models; NYHU 1 refers to the forest edge density (F.ED) model and NYHU 2 refers to the Stream + F.ED model. Blue bars indicate 95% credible intervals. Refer to Table 1 for species code definitions.

Based on the top ranked model(s), estimated mean probabilities of occupancy and turnover rates varied among species (Fig 4 and Table 4). MYLELUSE had the lowest estimated mean probability of occupancy (0.12) and NYHU had the highest estimated mean probability of occupancy (0.96; Fig 4). Both top ranked models for NYHU produced the same estimates of occupancy. Occupancy probabilities were lower for EPFULANO, LACI, MYAU, and NYHU in 2016 compared to 2015 and higher for DAIN, MYLELUSE, PESU, and TABR; however, estimates did not significantly differ between years for any species (Fig 4). Turnover rates ranged from 0.02 for NYHU (both top-ranked models) to 0.55 for MYLELUSE (Table 4) and 95% credible intervals were narrow for species with low turnover rates and wide for species with higher turnover rates (Table 4).

**Table 4:** Estimated turnover rates of species occupancy across South Carolina from May-July 2015 to May-July 2016.

Species	Turnover	Lower CI	Upper CI
**DAIN**	0.44	0.10	0.73
**EPFULANO**	0.06	0.01	0.17
**LACI**	0.42	0.06	0.80
**MYAU**	0.18	0.01	0.50
**MYLELUSE**	0.55	0.12	0.90
**NYHU 1**	0.02	5E-4	0.07
**NYHU 2**	0.02	6E-4	0.08
**PESU**	0.06	0.01	0.13
**TABR**	0.07	0.01	0.16

Estimates are based on the top ranked model for each species. Lower and Upper CI indicate 95% credible intervals. Refer to Table 1 for species code definitions.

**Fig 5:**
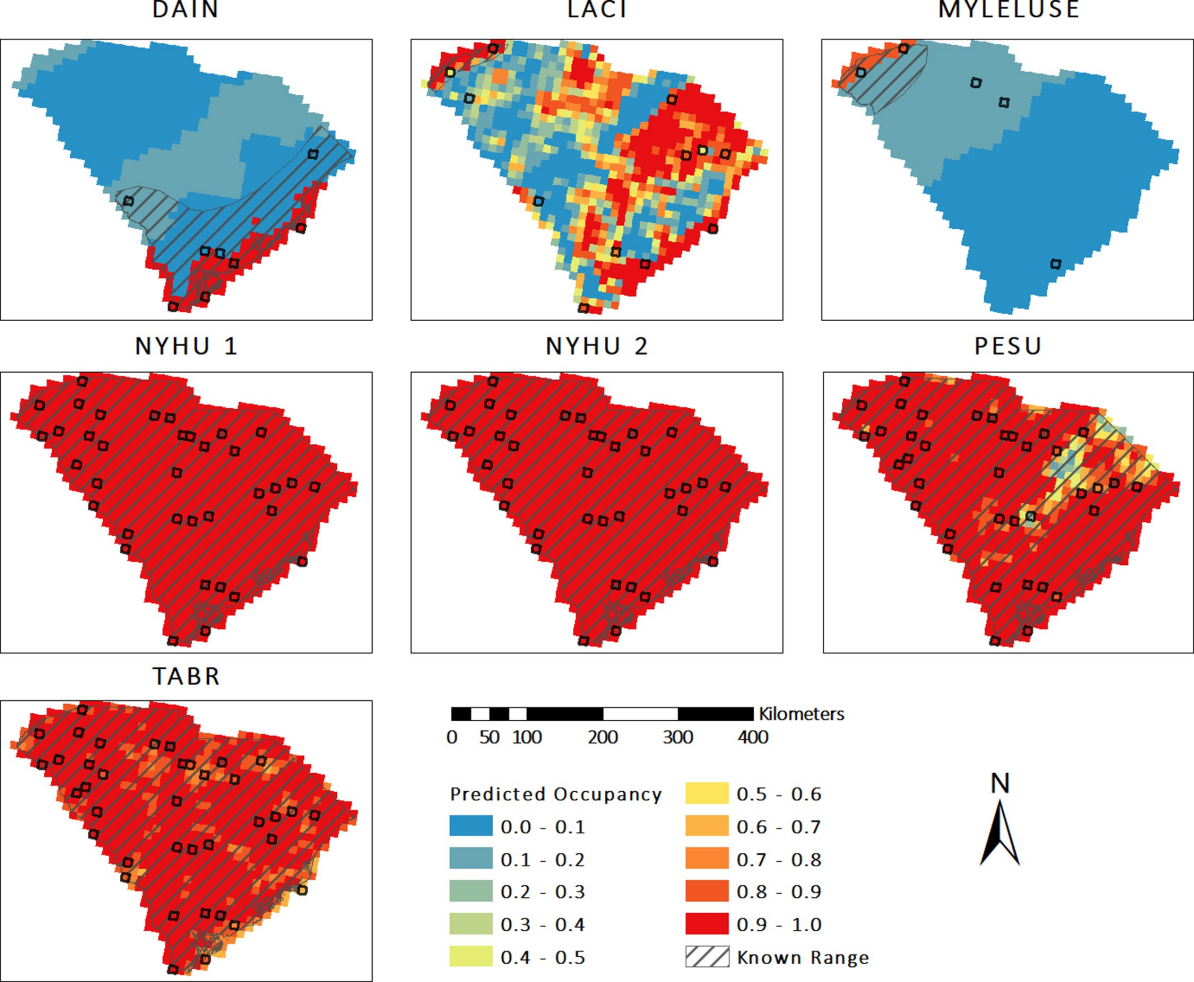
Predicted distribution maps for bat species across South Carolina. Distributions are based on effect estimates in the top-ranked occupancy model for each species, if non-null, and measures of environmental covariates in each cell. Black-outlined squares indicate cells where species were detected in 2015, 2016, or both years. Known summer ranges are based on Menzel et al. [43]. Refer to Table 1 for species code definitions.

We found that predicted distribution maps based on top-ranked occupancy models differed among species, but closely matched 2003 known ranges (Fig 5). DAIN and MYLELUSE, species for which Region was the top-ranked model, each had high probabilities of occupancy in ecoregions that are completely within their 2003 known ranges, and neither species had a predicted occupancy greater than 30% outside these regions. In 2003, the known distribution of NYHU was statewide and both models predicted occupancy rates greater than 90% statewide. The known distribution of TABR was also statewide, and the model predicted occupancy rates greater than 90% in most areas, except in areas with fewer roads, but rates were consistently above 50%. Although PESU known distribution was statewide, predicted occupancy rates were lowest, down to 15%, in areas with proportionally high agricultural land cover, and high throughout the rest of the state. LACI predicted occupancy rates were highest in the Blue Ridge region, which fully encompasses their 2003 known range. However, LACI occupancy was also high in much of the Southern Coastal Plain region and areas of other regions where stream length was high and forest edge density was low.

## Discussion

Similar to the diversity of threats facing bat populations, our findings highlight the diverse, and often species-specific, environmental factors that predict bat occupancy. In the southeast, where there is ongoing rapid urban and agricultural expansion [27], our results highlight the need to understand which factors are important to the occurrence of each species in designing appropriate conservation and management plans. In addition, WNS was only known to occur in a small northeastern portion of our study area during our research, but has expanded farther across the state and the southeastern US (https://www.whitenosesyndrome.org/). Our data can provide a critical baseline for future analyses of how land use changes influence bat distribution and habitat use over time [9].

Our results suggest that for some bat species, existing U.S. Level III Ecoregion delineations [40] incorporate many of the features we predicted to affect occupancy. Despite the Blue Ridge region making up only a small part of our study area and having only one priority cell (which led to a very wide 95% credible interval for the estimate in this region, Fig 2), we observed strong region-specific patterns in species occupancy and distribution for some species. For example, as we expected based on previous historical range maps [43], we found the highest probability of occupancy for DAIN in the Southern Coastal Plain region. By contrast, for MYLELUSE, we found a significantly higher mean estimated probability of occupancy in the Blue Ridge region than the Southeastern Plains region, as we expected based on their known occurrence in the Blue Ridge region [43]. However, we detected MYLELUSE in three of the five regions (although they were not detected in many cells throughout each region), including one detection in the Coastal Plain. Although this detection was far outside their known historical range [56], these findings are consistent with a similar study in North Carolina [63]. The low detections of MYLELUSE and detections across the state likely led to the relatively low predictive performance (AUC = 0.62) of our model and suggests further research is needed on these species. In contrast, we did not find support for a predicted effect of Region on LACI and MYAU occupancy. LACI appear to have more widespread summer distributions within our study area than expected (Fig 5) as has also been found in North Carolina [63]. MYAU were not detected in many cells within the regions they are thought to occupy, possibly due to their preference for forested wetlands [16,60] that occur in small isolated patches and therefore were not heavily surveyed. MYAU is more of a habitat specialist and typically roosts in tree cavities in wetland habitats and forages in riparian areas [16,60]. Due to its specific habitat requirements, measures of forested wetlands and stream lengths at the cell level may not have been appropriate for modeling MYAU occupancy, and a finer scale analysis may be more suitable. Thus, ecoregion may be a useful tool for determining management activities for some bat species, but not others, especially if species occur in regions in which they were not previously found or have specialized habitat requirements within the ecoregions in which they occur.

While our approach was sensitive for large-scale differences among ecoregions, it likely was not sensitive to site-specific habitat conditions that drive fine-scale habitat selection [35]. For example, apart from region, the only habitat covariate retained in our top candidate model set and for which parameter estimate confidence intervals did not overlap zero was a negative association between forest edge density and LACI occupancy. This selection pattern contradicts other studies that found LACI prefer to forage near forest edge and openings [14,64], but others have observed LACI select for forest interior [65] or exhibit no strong preference between forest edges and opening interiors [66]. Our observation must be considered within the context that LACI exhibit migratory behavior and the majority of individuals may move north, out of our study area during summer [42]. Further, LACI are high flyers [67] and do not always echolocate when they are commuting [68,69]. This may explain why we found a very low mean probability of detection (4.0%) [56], relatively high turnover rate (0.42; Table 4), and a low predictive performance for the top-ranked occupancy model. Although LACI occupancy estimates were relatively high (averaged about 49%), the higher turnover rate may be a further indication of transient individuals opportunistically using habitat as well as low detectability. Further investigation of LACI summer habitat use (e.g., radio tracking of individuals) may be needed to explain the potential effects of forest edge density on their habitat use. The null model likely ranked highest for EPFULANO because it occurs statewide [16], so landscape scale environmental covariates were not able to explain slight differences in occupancy. Although environmental models ranked higher than the null model for NYHU, PESU, and TABR, none of the effects were significant. These species, along with EPFULANO, occupied most of our study area and used a variety of habitats, which could explain why we failed to find significant effects of any landscape-scale environmental factors for these species. In a similar occupancy study in Missouri, Starbuck et al. [70] found greater effects of habitat conditions at the landscape scale than at more localized scales. However, they analyzed occupancy and assessed effects of habitat conditions at each survey point, while we analyzed occupancy and assessed habitat conditions at the scale of 100 km^2^ cells, and landscape factors that affect bat occupancy at points near cell edges may not be represented by cell-level metrics. Thus, conducting multi-scale analyses, with landscape occupancy at the cell level and site use at the level of stationary points and mobile transects, accompanied by more localized measures of habitat conditions, may produce results that better reflect bat species roosting and foraging preferences [35].

While we were not always able to gain insight into individual habitat conditions driving bat occupancy, our models were still useful and insightful for assessing distributions and will be valuable for assessing long-term patterns in landscape-level changes in bat populations. For instance, models for DAIN and MYLELUSE predicted high occupancy rates in regions where they were known to occur. Additionally, NYHU and TABR generally had high predicted occupancy rates statewide and are typically found throughout the state. PESU are positively associated with forest cover [18] and their predicted distribution was lower in areas with low forest coverage and high agricultural coverage. Many of the species which had models with low predictive performance also had low probabilities of detection [56] (Table 3), which could be an indication of biased AUC estimates due to false negatives (i.e., non-detection of species where they were actually present) [71]. Additionally, our data may not have been suitable for assessment with k-fold cross validation and AUC because we had a small sample size (i.e., 38 cells), which was divided into subsets of 25 cells for training data and 13 cells for testing data. Our top-ranked models for most species may therefore be sufficient for predicting landscape occupancy and could be used to guide future mist netting efforts and updating species range maps, even in cases where we determined covariate effects were not significant and found models had low predictive performance.

With longer-term monitoring, data from our two-year study can be utilized in future studies or serve as a baseline for additional analyses to assess landscape-level patterns and change in bat communities in the southeastern US. Overall, populations of some species may be declining in our study area [55] but it was not evident in our study at the landscape scale, likely due to only having two years’ of data. However, other studies at similar spatial scales detected changes in bat populations over time, but these studies were at longer temporal scales (eight to 15 years) [7,9,72]. Thus, if the monitoring we initiated is continued, it is likely that managers will be able to better detect impacts of WNS and other threats to bat populations in our study area [73]. Estimated turnover rates appear to be related to species detection probabilities, where species with high detection probabilities had low turnover rates, averaging about 5%, and species with lower detection probabilities had higher turnover rates, averaging about 40% [56] (Table 4). For example, high turnover rates and low probabilities of detection for DAIN and MYLELUSE could indicate false negatives (i.e., non-detection where species were actually present) each year. The higher turnover rate for LACI may be related to our potential detection of transient individuals, due to their migratory behavior [42]. However, species with higher turnover rates also had wide credible intervals, so these results should be interpreted with caution, and further study is needed.

Overall, by establishing standardized, statewide acoustic monitoring of bats, we found ecoregion and landscape-scale effects on bat occupancy, and provided baseline data which may be used to further evaluate bat species distributions and occupancy at a landscape scale in the face of WNS and rapid land use change. Future studies could utilize our predicted distribution maps and incorporate our data to conduct analyses at various spatial and temporal scales to potentially reveal additional effects of land cover variables on bat species occupancy, and changes in bat populations over time if monitoring is continued [8,73]. Results of our study and further analyses of our data (e.g., with finer scale habitat measures, and more years of data) can therefore increase ecological knowledge of bats and be used to inform conservation priorities [7–9], which is critical to the sustainability of bat populations due to the numerous threats they currently face.

## Supporting information

S1 Table Survey cell locations and number of survey hours in each cell. (DOCX)

S2 Table Top ranked detection model for each species. (DOCX)

S3 Table Results of Pearson’s correlation test for each tested occupancy covariate. (DOCX)

S4 Table Estimated *β* for intercepts and covariates in top ranked occupancy models for each species. (DOCX)
