# Supplementary material for: Variation in regional and landscape effects on occupancy of temperate bats in the southeastern U.S
Source: PLoS One. 2018 Nov 8;13(11):e0206857. doi: 10.1371/journal.pone.0206857 (PMC6226102; doi:10.1371/journal.pone.0206857)
Supplement: S1 Table — (DOCX) [file pone.0206857.s001.docx]

**S1 Table. Survey cell locations and number of survey hours in each cell.**

UTM coordinates (Zone 17S) of each grid cell center for surveys conducted across South Carolina. Survey cells were selected using the North American Bat Monitoring Program Master Sample. Hours represent the number of hours of surveys were conducted at stationary points and along mobile transects in 2015 and 2016 in each cell.

|  |  |  | 2015 Survey Hours | | 2016 Survey Hours | |
| --- | --- | --- | --- | --- | --- | --- |
| Cell No. | Northing | Easting | Mobile Transects | Stationary Points | Mobile Transects | Stationary Points |
| 124 | 3819877 | 501473 | 2.22 | 0 | 2.10 | 0 |
| 188 | 3750578 | 622849 | 1.95 | 0 | 1.93 | 52.00 |
| 348 | 3608184 | 569150 | 0 | 74.55 | 0 | 170.10 |
| 380 | 3710543 | 494008 | 2.08 | 0 | 2.07 | 0 |
| 444 | 3714100 | 535354 | 4.12 | 0 | 2.58 | 0 |
| 700 | 3845691 | 464827 | 3.12 | 40.0 | 2 | 40.07 |
| 892 | 3823849 | 604164 | 2.35 | 120.40 | 2.13 | 120.60 |
| 1116 | 3550413 | 488597 | 0 | 85.20 | 0 | 85.33 |
| 1148 | 3818958 | 378881 | 2.03 | 0 | 1.93 | 40.20 |
| 1212 | 3757239 | 644336 | 1.98 | 72.57 | 2.08 | 104.00 |
| 1404 | 3727655 | 384632 | 0 | 142.57 | 0 | 81.47 |
| 1771 | 3861240 | 365264 | 3.43 | 81.07 | 2.33 | 80.8 |
| 2107 | 3818470 | 317573 | 2.13 | 81.07 | 2 | 50.58 |
| 2140 | 3563799 | 531443 | 0 | 0 | 0 | 85.20 |
| 2172 | 3789567 | 374162 | 2.67 | 0 | 2.90 | 0 |
| 2236 | 3805405 | 529740 | 2.58 | 0 | 2.93 | 0 |
| 2748 | 3846787 | 393561 | 2.13 | 0 | 3.02 | 0 |
| 2795 | 3890613 | 369989 | 2.18 | 122.00 | 2.08 | 121.60 |
| 2940 | 3721175 | 618063 | 2.03 | 0 | 1.97 | 0 |
| 3164 | 3624267 | 531022 | 1.78 | 85.20 | 1.82 | 169.87 |
| 3196 | 3690472 | 429631 | 2.32 | 40.60 | 1.80 | 81.60 |
| 3260 | 3799159 | 569538 | 2.23 | 0 | 3.35 | 0 |
| 3868 | 3670855 | 426484 | 2.12 | 20.30 | 1.85 | 40.80 |
| 3900 | 3770903 | 493556 | 1.77 | 41.067 | 1.93 | 30.95 |
| 3964 | 3654472 | 658165 | 0 | 0 | 0 | 83.60 |
| 4220 | 3818318 | 511424 | 0 | 100.17 | 0 | 120.40 |
| 4284 | 3743913 | 601366 | 3.15 | 0 | 3.27 | 0 |
| 4444 | 3621135 | 550881 | 0 | 42.60 | 0 | 116.78 |
| 4540 | 3707421 | 513889 | 0 | 41.07 | 0 | 41.20 |
| 4796 | 3842577 | 484735 | 1.88 | 0 | 1.90 | 0 |
|  |  |  |  |  |  |  |

|  |  |  | 2015 Survey Hours | | 2016 Survey Hours | |
| --- | --- | --- | --- | --- | --- | --- |
| Cell No. | Latitude | Longitude | Mobile Transects | Stationary Points | Mobile Transects | Stationary Points |
| 4988 | 3821870 | 552816 | 3.08 | 0 | 3.42 | 0 |
| 5179 | 3825166 | 339057 | 2.68 | 81.07 | 1.73 | 101.17 |
| 5244 | 3806056 | 397214 | 2.05 | 0 | 2.05 | 0 |
| 5308 | 3752527 | 674174 | 2.55 | 0 | 2.62 | 41.67 |
| 5500 | 3757063 | 389347 | 1.62 | 40.60 | 1.62 | 40.67 |
| 5867 | 3859207 | 313892 | 3.55 | 152.75 | 2.63 | 121.80 |
| 5916 | 3620705 | 489720 | 0 | 0 | 1.67 | 0 |
| 6268 | 3781320 | 362638 | 2.03 | 40.33 | 2.05 | 40.33 |
