# Supplementary material for: Variation in regional and landscape effects on occupancy of temperate bats in the southeastern U.S
Source: PLoS One. 2018 Nov 8;13(11):e0206857. doi: 10.1371/journal.pone.0206857 (PMC6226102; doi:10.1371/journal.pone.0206857)
Supplement: S2 Table — (DOCX) [file pone.0206857.s002.docx]

**S2 Table. Top ranked detection model for each species.**

Predictive performance (AUC) of each model. “Clutter” categorizes the level of vegetation clutter at stationary points, “issue” denotes incomplete mobile surveys or stationary point equipment malfunctions, “date” is Julian day, “temp”, “RH”, and “wind” are average temperature, relative humidity, and wind speed during the survey periods, “rain” denotes the occurrence of rain during the survey periods, and “duration” is the length of the survey occasion in minutes. See Table 1 for species code definitions.

| **Species** | **Detection Model** | **AUC** |
| --- | --- | --- |
| **DAIN** | *clutter + issue* | 0.99 |
| **EPFULANO** | *clutter + issue + date + temp + RH + wind + rain* | 0.88 |
| **LACI** | *duration + temp* | 0.93 |
| **MYAU** | *duration + issue* | 0.90 |
| **MYLELUSE** | *clutter + issue* | 0.97 |
| **NYHU** | *issue + date + wind* | 0.73 |
| **PESU** | *duration + date* | 0.68 |
| **TABR** | *clutter + issue + RH* | 0.88 |
