# Supplementary material for: Variation in regional and landscape effects on occupancy of temperate bats in the southeastern U.S
Source: PLoS One. 2018 Nov 8;13(11):e0206857. doi: 10.1371/journal.pone.0206857 (PMC6226102; doi:10.1371/journal.pone.0206857)
Supplement: S3 Table — (DOCX) [file pone.0206857.s003.docx]

**S3 Table. Results of Pearson’s correlation test for each tested occupancy covariate.**

We considered an absolute r-value > 0.7 (gray background with white text) as an indication of significant correlation and did not include those covariates in the same models.

|  | **Region** | **Ag** | **Dev** | **Forest** | **F.Wet** | **Contagion** | **F.ED** | **F.Wet.ED** | **Stream** | **Pri** | **Sec** | **Ter** | **Qua** |
| --- | --- | --- | --- | --- | --- | --- | --- | --- | --- | --- | --- | --- | --- |
| **Region** | **1.00** | -0.26 | 0.16 | -0.36 | -0.12 | 0.02 | -0.21 | -0.08 | -0.37 | 0.20 | 0.00 | 0.18 | -0.38 |
| **Ag** | -0.26 | **1.00** | -0.11 | -0.30 | 0.25 | -0.53 | 0.04 | 0.36 | 0.15 | -0.16 | 0.04 | -0.05 | 0.19 |
| **Dev** | 0.16 | -0.11 | **1.00** | -0.13 | -0.24 | -0.07 | 0.01 | -0.19 | -0.05 | **0.72** | **0.83** | **0.95** | -0.20 |
| **Forest** | -0.36 | -0.30 | -0.13 | **1.00** | -0.62 | 0.37 | 0.68 | -0.66 | 0.62 | -0.08 | 0.02 | -0.14 | -0.18 |
| **F.Wet** | -0.12 | 0.25 | -0.24 | -0.62 | **1.00** | -0.53 | -0.43 | **0.94** | -0.30 | -0.20 | -0.27 | -0.19 | 0.48 |
| **Contagion** | 0.02 | -0.53 | -0.07 | 0.37 | -0.53 | **1.00** | -0.25 | -0.63 | -0.04 | 0.02 | -0.17 | -0.19 | -0.28 |
| **F.ED** | -0.21 | 0.04 | 0.01 | 0.68 | -0.43 | -0.25 | **1.00** | -0.41 | 0.59 | -0.14 | 0.21 | 0.01 | -0.03 |
| **F.Wet.ED** | -0.08 | 0.36 | -0.19 | -0.66 | **0.94** | -0.63 | -0.41 | **1.00** | -0.24 | -0.17 | -0.16 | -0.11 | 0.52 |
| **Stream** | -0.37 | 0.15 | -0.05 | 0.62 | -0.30 | -0.04 | 0.59 | -0.24 | **1.00** | -0.16 | 0.15 | -0.04 | 0.00 |
| **Pri** | 0.20 | -0.16 | **0.72** | -0.08 | -0.20 | 0.02 | -0.14 | -0.17 | -0.16 | **1.00** | 0.58 | 0.68 | -0.15 |
| **Sec** | 0.00 | 0.04 | **0.83** | 0.02 | -0.27 | -0.17 | 0.21 | -0.16 | 0.15 | 0.58 | **1.00** | **0.81** | -0.19 |
| **Ter** | 0.18 | -0.05 | **0.95** | -0.14 | -0.19 | -0.19 | 0.01 | -0.11 | -0.04 | 0.68 | **0.81** | **1.00** | -0.22 |
| **Qua** | -0.38 | 0.19 | -0.20 | -0.18 | 0.48 | -0.28 | -0.03 | 0.52 | 0.00 | -0.15 | -0.19 | -0.22 | **1.00** |
