# Supplementary material for: Variation in regional and landscape effects on occupancy of temperate bats in the southeastern U.S
Source: PLoS One. 2018 Nov 8;13(11):e0206857. doi: 10.1371/journal.pone.0206857 (PMC6226102; doi:10.1371/journal.pone.0206857)
Supplement: S4 Table — (DOCX) [file pone.0206857.s004.docx]

**S4 Table. Estimated *β* for intercepts and covariates in top ranked occupancy models for each species.**

Coefficient 95% credible intervals (Lower and Upper CI), and their convergence values (R-hat) are shown. See Table 1 for species code definitions.

| **Species** | **Parameter** | **β estimate** | **Lower CI** | **Upper CI** | **R-hat** |
| --- | --- | --- | --- | --- | --- |
| **DAIN** | Intercept | -2.07 | -4.87 | 2.80 | 1.01 |
|  | Blue Ridge | 0.00 | 0.00 | 0.00 | 0.00 |
|  | Mid-Atlantic Coastal Plain | -22.57 | -70.25 | 3.92 | 1.00 |
|  | Piedmont | -25.68 | -70.22 | -0.85 | 1.00 |
|  | Southeastern Plains | 0.02 | -5.52 | 4.10 | 1.00 |
|  | Southern Coastal Plain | 27.82 | 3.96 | 71.43 | 1.00 |
| **LACI** | Intercept | 0.14 | -2.00 | 3.26 | 1.00 |
|  | Stream | 2.17 | -0.61 | 4.69 | 1.00 |
|  | F.ED | -2.89 | -4.84 | -0.49 | 1.00 |
| **MYLELUSE** | Intercept | 1.81 | -2.39 | 4.83 | 1.00 |
|  | Blue Ridge | 0.00 | 0.00 | 0.00 | 0.00 |
|  | Mid-Atlantic Coastal Plain | -27.46 | -72.06 | -3.25 | 1.00 |
|  | Piedmont | -3.32 | -7.06 | 1.19 | 1.00 |
|  | Southeastern Plains | -28.03 | -72.89 | -3.97 | 1.00 |
|  | Southern Coastal Plain | -24.56 | -70.47 | 0.40 | 1.00 |
| **NYHU 1** | Intercept | 4.06 | 2.49 | 4.96 | 1.00 |
|  | F.ED | -0.86 | -2.94 | 1.57 | 1.00 |
| **NYHU 2** | Intercept | 4.17 | 2.68 | 4.97 | 1.00 |
|  | Stream | 0.15 | -2.08 | 2.57 | 1.00 |
|  | F.ED | -0.88 | -3.16 | 1.80 | 1.00 |
| **PESU** | Intercept | 4.41 | 3.14 | 4.98 | 1.00 |
|  | Ag | -1.20 | -3.36 | 1.58 | 1.00 |
|  | Dev | 1.38 | -1.07 | 4.32 | 1.00 |
|  | For | 0.38 | -2.15 | 2.77 | 1.00 |
|  | Qua | 1.48 | -0.62 | 4.22 | 1.00 |
| **TABR** | Intercept | 4.16 | 2.69 | 4.97 | 1.00 |
|  | Pri | 1.06 | -1.85 | 4.46 | 1.00 |
|  | Sec | 1.38 | -0.75 | 3.91 | 1.00 |
|  | Qua | 1.37 | -0.53 | 4.00 | 1.00 |
